# Hicks’ Mercantile College

**Published:** 1863-11

**Authors:** 


Hicks' Mercantile College.—We would call the attention of those who have sons to educate, to the winter term of this institution, where those who prefer to profit by their knowledge, can do so by attending this business college, which is one of the most extensive in its practice, having for its Preceptor, a practical accountant thoroughly acquainted in all the departments of trade. Graduation in this institution is a passport to desirable locations in the most respectable mercantile houses in the city.

